# Description of Common Ailments and Nonprescription Medications Found in Medication Reviews for People With Intellectual Disability

**DOI:** 10.1111/jir.13243

**Published:** 2025-04-24

**Authors:** Chelsea Felkai, Jamie‐Lee Carew, David Newby, Hayley Croft

**Affiliations:** ^1^ School of Biomedical Sciences and Pharmacy, College of Health Medicine and Wellbeing The University of Newcastle Callaghan New South Wales Australia

**Keywords:** common ailments, intellectual disability, medication reviews, nonprescription medication

## Abstract

**Background:**

People with intellectual disability (ID) are more susceptible to experiencing minor health issues. This research describes the common ailments and nonprescription medications found in people with ID who have had a medication review performed by a credentialed pharmacist in Australia.

**Aims:**

The aims of this research were to (i) describe the common ailments found within people with ID and (ii) identify and quantify the nonprescription medications documented in medication reviews for people with ID.

**Method:**

This research conducted a retrospective analysis of medication review reports and referrals from credentialed pharmacists who have performed a medication review for a person with ID between January 2020 and January 2024.

**Results:**

A total of 80 responses and reports were obtained. The average age of the person with ID was 52 years. On average, each medication review listed 6.6 common ailments and 8.0 nonprescription medications. The highest number of nonprescription medications listed for a single individual was 26.

**Conclusion:**

This research is the first to exclusively examine common ailments and nonprescription medications found in people with ID through medication reviews. Further research is needed to confirm study findings revealing a potentially high occurrence of common ailments and nonprescription medication use in this population compared to other similar populations and notable polypharmacy for nonprescription medications.

## 1 Introduction

People with intellectual or developmental disability (ID) account for approximately 6.5% of the 4.4 million Australians with disability (Dew and Gaskin 2020). People with ID are at an increased risk of experiencing common ailments, typically defined as health conditions that can be self‐identified and managed through self‐care or with nonprescription medications (International Pharmaceutical Federation (FIP) 2023) compared to the general population (Author 2021). Common ailments can be associated with the developmental disability itself; for example, anatomical changes to the gastrointestinal tract and poor mobility can result in a higher risk of GORD, malnutrition, constipation, incontinence and urinary tract infections (UTIs). Musculoskeletal issues are especially more common in people with cerebral palsy due to increased scoliosis, hip problems and osteoporosis (Author 2021). People with ID often need support in undertaking hygiene practices, which can lead to a higher prevalence of viral, bacterial and fungal infections (Author 2021). The increased prevalence of physical (Kinnear et al. 2018; Liao et al. 2021) and psychiatric (Hughes‐McCormack et al. 2017; Bratek et al. 2017; Dias et al. 2013) conditions compared to the general population also increases the risk of common ailments. The higher rates of physical health conditions such as diabetes (Liao et al. 2021; MacRae et al. 2015) and hypothyroidism (Liao et al. 2021) in people with ID also increase the risk of common ailments including thrush, tinea, dry skin, joint pain and UTIs (Author 2019a; Author 2019b). Medications commonly used by people with ID including antipsychotics, antidepressants and anticholinergic medicines carry adverse effects such as constipation (O'Dwyer et al. 2016), dry eye and mouth, weight gain and gastro‐oesophageal reflux disease (Matson and Mahan 2010). Pain or discomfort due to physical ailments, including gastrointestinal conditions, inflammatory or infectious diseases, cardiac disease, headache and orofacial and eye conditions, can play a role in challenging behaviours in people with ID (de Winter et al. 2011).

The presence of common ailments increases the potential for polypharmacy as additional medications are often used to treat these ailments. Many of these medications can be obtained without a prescription, along with other vitamin and herbal supplements and products without an active ingredient such as creams. Nonprescription medicines are also often used in the treatment of more serious or chronic conditions, such as calcium and vitamin D in the management of osteoporosis and low dose aspirin used in the prevention of cardiovascular events. Understanding the use of nonprescription medications and supplements remains challenging due to the reliance on patient recall, varying interpretations of what medications or supplements should be included, or the use of prescribing software that may not capture over the counter medications typically purchased without a prescription. As such, there is relatively little known about common ailments and nonprescription medication use in research (Liao et al. 2021; Robertson et al. 2015).

Government funded home medicines review (HMR) provided in Australia requires a general practitioner (GP) to provide a referral to a credentialed pharmacist for a patient living in the community whom the GP believes is currently experiencing, or at risk of experiencing, a medication misadventure (Pharmacy Programs Administrator 2018). The HMR service must be performed by a pharmacist who has undertaken a nationally recognised training course to become credentialed and involves reviewing available clinical documents in combination with an interview with the patient and carers in the patient's home. This combination of clinical documents and interview with the patient helps to develop a best possible medication history (NSW Clinical Excellence Commission n.d.). The outcome of the HMR is threefold: education provided to the patient and caregiver, a report developed for the GP with a comprehensive list of medications the patient is taking and any issues and recommendations for optimal medication use and a medication management plan developed between the patient and GP based on the recommendations provided by the pharmacist's report (Bergin et al. 2020). This research identifies the type and occurrence of common ailments and nonprescription medications found in people with ID who have had a medication review performed by a credentialed pharmacist in Australia since 2020.

## 2 Methods

Credentialed pharmacists were invited to participate in the study if they had conducted a government funded HMR service for a person with ID living within the community since January 2020. Medication reviews performed on residents within aged care facilities were also excluded as it falls under a separate service. Invitations to participate were addressed to credentialed pharmacists who had performed a medication review since 2020, for a person with ID living in the community, and included an attachment of the participant information statement and a survey link. These invitations were disseminated on the following private, pharmacist‐specific Facebook groups: ‘Pharmacists Optimising Medicines in People with Intellectual Disability and Autism (POMPIDA)’, ‘Consultant Pharmacists of Australia’ and ‘Pharmaceutical Society of Australia (PSA) Early Career pharmacists’. Email invitations were also disseminated through pharmacist member organisations including the PSA, the Australian Association of Consultant Pharmacy (AACP) and the Newcastle and Hunter Valley Pharmacist Association (NHVPA). Snowball recruitment was also used as those who received the email were invited to forward the email on to other pharmacists who may be eligible to participate.

The survey link led the participant to an online survey that asked the participant to upload a redacted medication review report and referral and answer a series of 10 questions related to the medication review. The questions included demographic information regarding the patient involved in the medication review and details on the service itself. The participant could complete the questionnaire up to 10 times for 10 different medication reviews and received a $20 honorarium for each completed questionnaire. A redaction protocol was provided to participants to support them in fully redacting the medication review reports and referrals prior to uploading them into the survey. One research investigator (C.F.) reviewed the uploaded reports and referrals, permanently deleting any that were not fully redacted prior to data extraction.

A list of common ailments was derived initially from Australian and UK context using the ‘Community Pharmacy: Symptoms, Diagnosis and Treatment’ by David Newby and Paul Rutter (Rutter and Newby 2016) and further expanded on using the Federation of International Pharmacists (FIPs) ‘Pharmacists‐Led Common Ailments Scheme: A Global Intelligence Report’ (International Pharmaceutical Federation (FIP) 2023). Nonprescription medications comprised all products listed on the medication review report that could be purchased without a prescription in Australia. The Poisons Standard and Scheduling of Medicines and Compounds (SUSMP) is an Australian standard used as a legislative instrument for the uniform scheduling of substances, which determines the supply of medications to the public. Substances that were classified as unscheduled, which can be obtained at any retailer; Schedule 2 products that can only be obtained at a community pharmacy; and Schedule 3 products that can only be obtained without a prescription by speaking with a pharmacist were included in the list of nonprescription medicines. Medication review reports were read through, and the list of common ailments and nonprescription products was transcribed into an excel document for each report.

## 3 Analysis

Questionnaire data was downloaded directly into a spreadsheet and included demographic information related to the medication review and the living arrangement of the person with ID. Nonprescription products were extracted from each report and then coded into a spreadsheet using the Anatomical Therapeutic Chemical (ATC) classification (WHO Collaborating Centre for Drug Statistics Methodology 2009). Common ailments were transcribed from each report and categorised using the International Classification of Diseases (ICD‐11) (World Health Organization 2022); both classification systems were developed by the World Health Organization. An initial reviewer (C.F.) transcribed and coded all 80 HMR reports, while a second reviewer (J.L.‐C.) coded and entered data for 20 of the reports. The results from both reviewers were compared and discussed within the research team to ensure reliability in data analysis. Ethics approval was granted for this project by the University of Newcastle Human Research Ethics Committee, protocol H‐2022‐0043.

## 4 Results

### 4.1 Overall Results

Seventeen credentialed pharmacists uploaded a total of 80 HMR reports and completed the corresponding questionnaire data. The questionnaire data shows an almost equal ratio of men (49%) and women (51%) whose HMR data were used for this study. The average age of the participants was 52 years, with the youngest being 12 years of age and the oldest being 76 years of age at the time of the review. Each medication review listed between six and seven common ailments and an average of eight nonprescription medications (Table 1).

**TABLE 1:** Overview of results from medication review study.

Demographic	Parameter	Study results
Gender	Female	51%
	Male	49%
Age (years)	Mean	52 years
	Range	12–76 years
State/territory	New South Wales	47.5%
	Queensland	20%
	Victoria	12.5%
	South Australia	12.5%
	Western Australia	7.5%
Type of disability	Developmental delay	26.9%
	Learning disability	21.8%
	Autism spectrum disorder	12.8%
	Intellectual disability	10.3%
	Trauma (e.g., acquired brain injury)	7.7%
	Cerebral palsy	6.4%
	Trisomy 21 (Down's syndrome)	5.1%
	Angelman's syndrome	3.8%
	Mental health (bipolar/schizophrenia)	3.8%
	Other	15.4%
	Unknown	19.2%
Living arrangement	In a supported independent living arrangement, or ‘group home’, with a full‐time paid carer	89.74%
	In their own home with an informal carer (such as a family member)	5.13%
	In their own home with a full‐time paid carer	3.85%
	In their own home with periodic assistance from a carer	1.28%

Investigation	Parameter	Results (per review)
Common ailments	Mean	6.6
	Range	1–23
Nonprescription medications	Mean	8.0
	Range	1–26

### 4.2 Common Ailments and Chronic Disease

**FIGURE 1:**
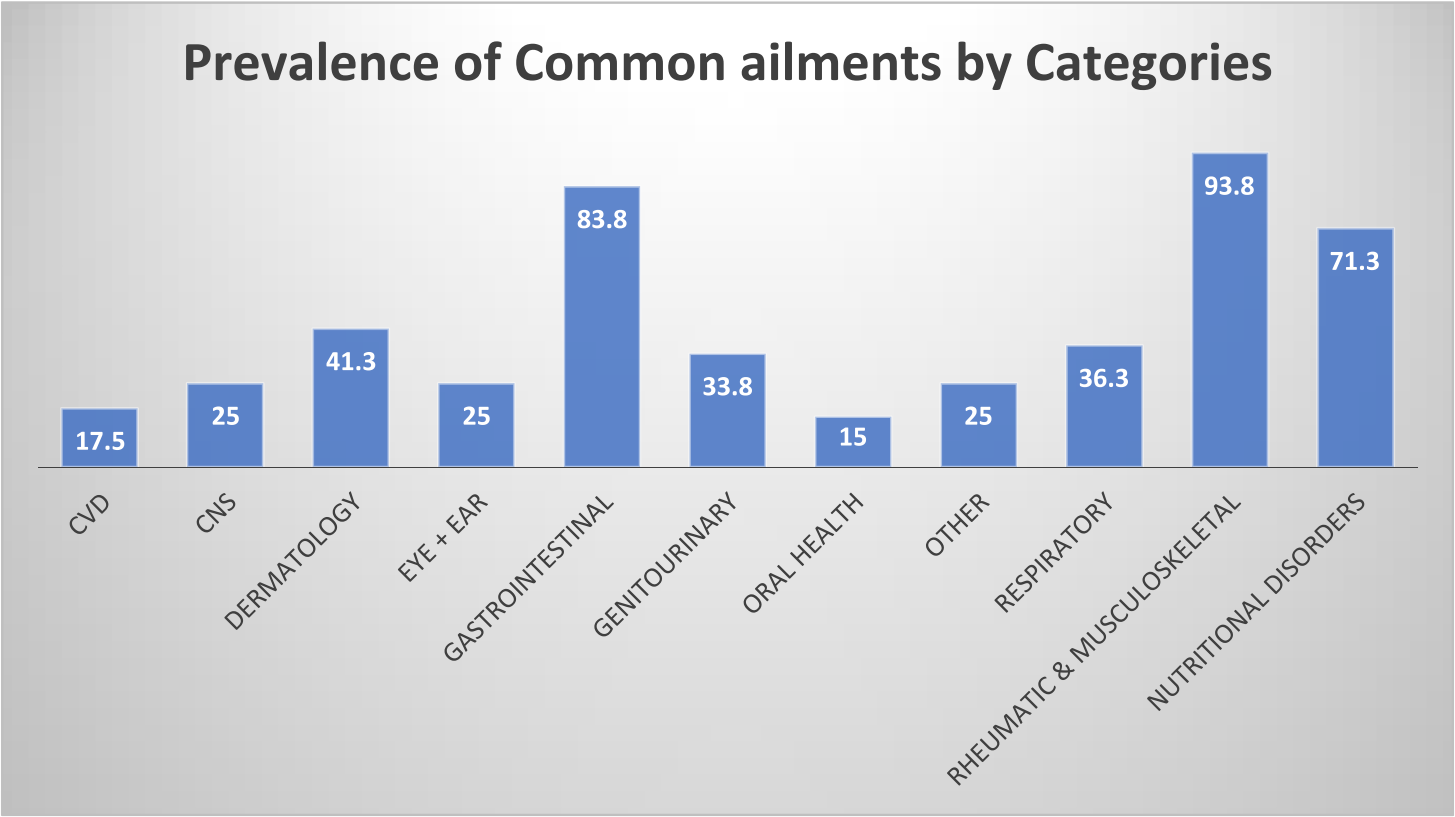
Prevalence of categories of common ailments identified in people with ID during medication review by pharmacist.

The most common category of ailments found in this study was rheumatic and musculoskeletal, followed by gastrointestinal conditions and nutritional disorders (Figure 1). For some categories such as gastrointestinal conditions, rheumatic and musculoskeletal, nutritional disorders, dermatology and respiratory, it was common for an individual to experience more than one condition within the same category. A full list of the common ailments and percentage of the study population listed with them can be found in Appendix 1.

Although the most prevalent category of common ailment was rheumatic and musculoskeletal conditions, when looking at specific conditions within the categories, constipation was the most prevalent common ailment, followed by nonspecific pain and fever and then by GORD (Table 2).

**TABLE 2:** Top 10 common ailments identified in medication review report ranked by prevalence.

Rank	Common ailment	(*n*)	%
1	Constipation	58	72.5
2	Infrequent pain/fever	54	67.5
3	Dyspepsia and GORD	44	55
4	Vitamin D deficiency/prevention	33	41.3
5	Chronic pain	30	37.5
6	Allergic rhinitis and rhinosinusitis	19	23.8
7	Urinary incontinence	19	23.8
8	Asthma	17	21.3
9	Dry skin	17	21.3
10	Sleep disorder	16	20

### 4.3 Nonprescription Medications

**FIGURE 2:**
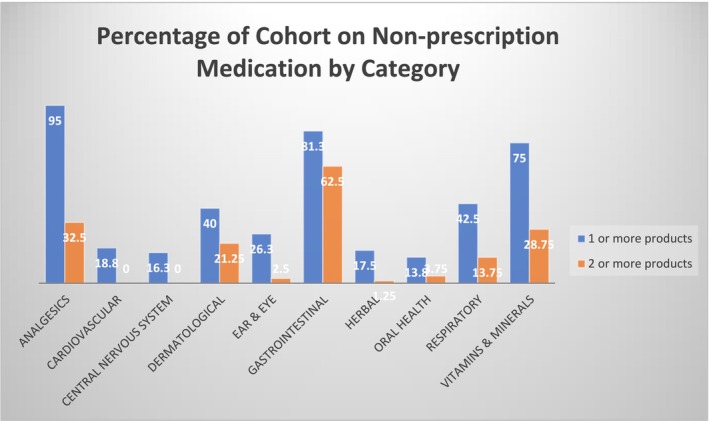
Percentage of cohort using a nonprescription product by category compared with those using two or more products in the same category identified in people with ID during medication reviews by pharmacists.

A total of 640 nonprescription medications were documented in the medication reviews. Regular nonprescription medications made up 56.9% of the total of nonprescription medicines, while the remaining 43.1% were used when required (PRN). Figure 2 shows that analgesics are the most prevalent category of nonprescription medicines, followed by gastrointestinal and vitamins and minerals.

Paracetamol was found in 92.5% of reviews (Table 3), although only 27.5% were using it regularly. Laxatives made up most of the nonprescription medications, with 72.5% of reviews including a laxative in the medication list. A full list of the nonprescription medicines and the percentage of the study population taking them can be found in Appendix 1.

**TABLE 3:** Top 10 nonprescription medicines identified in the medication review report ranked by prevalence.

Rank	Nonprescription medicine	(*n*)	%
1	Paracetamol (total)		92.5%
2	Osmotic laxatives		71.3%
3	Vitamin D		56.3%
4	Emollients and protective creams/ointments		53.8%
5	Proton pump inhibitors		50%
6	Fixed‐dose combination stool softeners and stimulants		45%
7	Bulk‐forming laxatives		27.5%
8	Salbutamol		23.8%
9	Less‐sedating antihistamines		22.5%
10	Ibuprofen		20%

### 4.4 Polypharmacy

Polypharmacy has commonly been defined as five or more regular medications (Masnoon et al. 2017), while hyperpolypharmacy refers to the use of 10 or more medications (Palmer et al. 2019). Using these definitions, 40% of people with ID had polypharmacy related to their regular nonprescription products, while 7.5% experienced hyperpolypharmacy. When PRN medications were included, polypharmacy was seen in 78% of people with ID, and hyperpolypharmacy occurred in 36% of this cohort. Multiple products used within the same category frequently occurred for gastrointestinal, dermatological, vitamin, respiratory and analgesic products, as shown in Table 4. Gastrointestinal products were a common source of multiple medication use, with 62.5% of the cohort using two or more gastrointestinal medications, and some individuals on up to four different laxative products.

**TABLE 4:** Average number of products and maximum number of products used per person by category of nonprescription medication.

Nonprescription medication category	Number of products per person with condition	Maximum number of products used by a single individual
Gastrointestinal	2.7	6
Dermatological	2.5	7
Vitamins	1.6	5
Respiratory	1.5	5
Analgesic	1.4	4

## 5 Discussion

This research is the first to review and describe the common ailments and nonprescription medications found in people with ID through medication review performed by pharmacists in Australia. Previous research has identified pain and constipation to be significant issues for people with ID; however, rates identified in this research exceed international prevalence rates where data exists. Difficult‐to‐manage conditions such as pain and the use of analgesics have gained attention for people with ID due to the increasing evidence of underdiagnosis and undertreatment (McGuire et al. 2010; Boerlage et al. 2013a). International studies of chronic pain in people with ID show prevalence rates of 13%–18% (McGuire et al. 2010; Walsh et al. 2011; Boerlage et al. 2013b), while this research identified a combination of chronic pain and osteoarthritis prevalence of 32.5% for people with ID. Paracetamol was used regularly by 27.5% of people with ID, compared with 8.8% of the older Australian population (Goh et al. 2009). This could be an indication of increasing awareness of the need to manage pain in people with ID who may not be able to communicate symptoms of pain effectively. It may also be because 89.7% of the cohort resided in supported independent living, where pain management may have been provided regardless of whether pain symptoms were present or not. The cohort's living arrangement in group homes may also be why three quarters of the population were on a vitamin supplement, mostly vitamin D, and nutritional deficiencies were identified in two‐thirds of reviews.

A notable finding in this research was the level of common ailment multimorbidity, with reviews listing an average of 6.6 conditions per person with ID. The multimorbidity is accompanied by a high level of nonprescription medication use, with over 90% reporting taking at least one product and high rates of nonprescription medicine polypharmacy. Due to the lack of research in nonprescription medications for people with ID, studies of those living in the community over 60 years were used as a reference, as polypharmacy is well documented in the older population (Davies et al. 2020). Two Australian studies have been published that investigated nonprescription medication use in older people. The Australian Longitudinal Study of Ageing is a population‐based study by Goh et al., which found 35.5% of older adults were using a nonprescription medicine (Goh et al. 2009), while a similar‐sized study by Simons et al. found 69.3% (Simons et al. 1992). A systematic review by Jerez‐Roig et al. found that people over the age of 60 years were on an average of 0.3–2.4 nonprescription medicines per person compared with this study's average of eight nonprescription medications per person with ID (Jerez‐Roig et al. 2014). One Canadian study identified rates of 20.5% prevalence for five or more regular nonprescription medications in an older population with frailty (Harris et al. 2022), while the results from this research identified double the prevalence (40%) of people with ID taking five or more products. The addition of PRN nonprescription medications increases the burden of polypharmacy to 78%, as well as increasing the complexity of managing the use of nonprescription medications and the risk of medication‐related harm. These findings demonstrate that potentially excessive rates of polypharmacy for nonprescription medicines might be occurring in people with ID even when compared to a population that is well documented as experiencing high levels of comorbidities and nonprescription medication use. Nonprescription medications and health products can also contribute to nonadherence to prescription medication in people with multimorbidity (Anoopkumar‐Dukie et al. 2020), and a study by Yap et al. showed that even short‐term medications can affect adherence in elderly populations (Yap et al. 2016).

This study highlights that people with ID face a potentially significant issue in managing common ailments and nonprescription medications in an increasingly complex health care environment with limited access to GPs. It has been established in national and international research that people with ID are more likely to have a potentially preventable hospital admission than the general population (Balogh et al. 2010; Dunn et al. 2018; Glover et al. 2020; Weise et al. 2021). Australian data shows some of the highest rates of potentially preventable hospital admissions include common ailments that could be treated in the primary care setting, such as nutritional deficiencies; UTIs, ear, nose and throat infections and dental conditions (Health Stats NSW 2022). In addition, the rate of hospitalisation is 3.5 times higher for people with ID than the general population, and this difference is mostly found primarily in acute and vaccine‐preventable conditions (Weise et al. 2021). One of the leading causes of patient harm in health care is medication errors, and medication categories identified in this study to have high levels of usage and to be involved in polypharmacy, such as gastrointestinal, musculoskeletal and respiratory medications, are main drug classes associated with medication‐related harm (Organization WH 2024). By understanding the type and prevalence of common ailments and nonprescription medications used by people with ID, pathways for accessible treatment within the primary care setting can be explored.

### 5.1 Strengths and Limitations

The low sample size of 80 HMRs limits the conclusions that can be drawn from this data set, particularly related to prevalence as the sample size was not representative of the people with ID population living within Australia. There are no data available to determine how many HMRs are completed annually within the Australian population, and of those how many were provided to people with ID in order to determine if it is representative of those who have received a HMR. However, the 80 HMRs used in this study were derived from 17 different pharmacists and five different states of Australia helping to reduce bias from the small sample size. Medication reviews typically tend to focus on medications, and as such, common ailments that did not require medications may have been inconsistently documented. This may have led to the underrepresentation of conditions such as incontinence, obesity, oral health and smoking cessation within this research. The focus on medications, however, means the data used for nonprescription medicines was likely of high quality, as the creation of a best possible medication history requires a thorough investigation to produce the list of what a patient is currently taking. In some instances, the listing of a condition was difficult to appropriately categorise, as a medication may have been indicated ‘for rash’, ‘for pain’ or ‘for bones’. Some common ailments included require complex care, such as chronic pain; however, due to the use of nonprescription medications such as simple analgesics in this condition, this condition was also included.

## Conclusion

This research is the first to describe the common ailments and nonprescription medications found in people with ID through medication review performed by pharmacists in Australia. Further research is needed to confirm study findings revealing a potentially high occurrence of common ailments and nonprescription medication use in this population compared to other similar populations and notable polypharmacy for nonprescription medications. This study highlights a potentially elevated risk of common ailment multimorbidity and polypharmacy for nonprescription medications, which should be a focus for future study in people with ID.

## Ethics Statement

Ethics approval was granted for this project by the University of Newcastle Human Research Ethics Committee, protocol H‐2022‐0043.

## Conflicts of Interest

The authors declare no conflicts of interest.

## Supporting information


**Table S1.** Percentage of study population with common ailment condition by category.
**Table S2.** Percentage of study population taking a nonprescription medication by category.

## Data Availability

The data that supports the findings of this study are available in the supporting information of this article
