# Supplementary material for: Description of Common Ailments and Nonprescription Medications Found in Medication Reviews for People With Intellectual Disability
Source: J Intellect Disabil Res. 2025 Apr 24;69(7):613–20. doi: 10.1111/jir.13243 (PMC12198093; doi:10.1111/jir.13243)
Supplement: Supplementary file 1 — Table S1. Percentage of study population with common ailment condition by category. Table S2. Percentage of study population taking a nonprescription medication by category. [file JIR-69-613-s001.docx]

| *CATEGORY* | *CONDITION* | *PERCENTAGE OF STUDY POPULATION* |
| --- | --- | --- |
| *Rheumatic and musculoskeletal conditions* | Infrequent pain or fever | 67.5% |
|  | Non-specified chronic pain | 17.5% |
|  | Osteoarthritis | 15.0% |
|  | Acute pain | 10.0% |
|  | Muscle cramps | 8.8% |
|  | Neuropathic pain | 2.5% |
|  | Osteopenia | 2.5% |
| *Gastrointestinal conditions* | Constipation | 72.5% |
|  | Gastro-oesophageal reflux disease | 55.0% |
|  | Diarrhoea | 8.8% |
| *Nutritional disorders* | Underweight | 10.0% |
|  | Obesity | 8.8% |
|  | Actual or potential vitamin deficiency (TOTAL) | *71.3%* |
|  | Vitamin D deficiency | 41.3% |
|  | Bone deficiency prevention | 21.3% |
|  | Iron deficiency | 17.5% |
|  | Vitamin B12 deficiency | 8.8% |
|  | Calcium deficiency | 2.5% |
|  | Folic acid deficiency | 2.5% |
|  | Vitamin B1 | 2.5% |
| *Dermatological conditions* | Dry skin | 21.3% |
|  | Fungal skin infections | 18.8% |
| Respiratory conditions | Allergic rhinitis | 23.8% |
|  | Asthma | 21.3% |
|  | Cold and flu | 5.0% |
| *Genitourinary* | Urinary incontinence | 23.8% |
|  | Dysuria and cystitis | 7.5% |
|  | Dysmenorrhoea | 5.0% |
|  | Contraception | 5.0% |
|  | Vaginal acidity | 3.8% |
| *CNS* | Insomnia | 20.0% |
|  | Headache and migraine | 5.0% |
|  | Motion sickness | 5.0% |
| *eye and/or ear* | Dry eye | 13.8% |
|  | Ear wax impaction | 10.0% |
| *CVD and other conditions* | Cardiovascular (CVD) was included to capture low dose aspirin intake | 17.5% |
|  | Smoking cessation | 1.3% |
|  | Wound management | 3.8% |
|  | Percutaneous endoscopic gastrostomy (PEG) | 3.8% |

**BREAKDOWN OF COMMON AILMENT AND NON-PRESCRIPTION MEDICINE USAGE FOUND IN MEDICATION REVIEWS WITH PEOPLE WITH INTELLECTUAL DISABILITY**

Table 1. Percentage of study population with common ailment condition by category.

| *CATEGORY* | *NON-PRESCRIPTION MEDICINE* | *PERCENTAGE OF STUDY POPULATION* |
| --- | --- | --- |
| *Analgesic products* | Paracetamol (TOTAL) | 92.5% |
|  | Paracetamol (PRN/when required) | 68.8% |
|  | Paracetamol (regular use) | 27.5% |
|  | Ibuprofen | 20.0% |
|  | Topical pain relief including anti-inflammatories and rubefacients | 17.5% |
| *Gastrointestinal products* | Osmotic laxatives | 71.3% |
|  | Fixed-dose combination stool softeners and stimulants | 45.0% |
|  | Bulk-forming laxatives | 27.5% |
|  | Stimulant | 5.0% |
|  | Stool softener | 5.0% |
|  | Proton pump inhibitors | 50.0% |
|  | Antacids | 13.8% |
| *Vitamins and Minerals* | Vitamin D | 56.3% |
|  | Iron | 16.3% |
|  | Calcium | 12.5% |
|  | Magnesium | 10.0% |
|  | Vitamin B12 | 9.0% |
|  | Vitamin C | 5.0% |
|  | Folic acid | 4.0% |
|  | Multivitamins | 4.0% |
|  | Vitamin B1 | 3.0% |
| *Dermatological products* | Emollients and protectives | 53.8% |
|  | Topical antifungal creams | 18.8% |
|  | Topical corticosteroids | 12.5%, |
|  | Antifungal and corticosteroid combination cream | 6.0% |
| *Respiratory medications* | Salbutamol | 23.8% |
|  | Less-sedating antihistamines | 22.5% |
|  | Nasal sprays | 12.5% |
|  | Cough and cold products | 5.0% |
| *Eye and Ear products* | Lubricating eye drops | 16.3% |
|  | Ear wax softeners | 10% |
| *Oral health products* | Chlorhexidine mouthwash, gel, or toothpaste. | 10% |
|  | Flouride toothpaste | 2.5% |
|  | Ulcer product | 2.5% |
|  | Anti-infective (cold sore/oral thrush products) | 2.5% |
| *Herbal products* | Fish Oil | 10.0% |
|  | Probiotics | 5.0% |
| *Other medications* | Low dose aspirin | 17.5% |
|  | Wound products | 13.8% |
|  | Nutritional supplements | 12.5% |

Table 2. Percentage of study population taking a non-prescription medication by category.
